# Genome-Wide Association Study of Root System Architecture in Maize

**DOI:** 10.3390/genes13020181

**Published:** 2022-01-28

**Authors:** Bin Wu, Wei Ren, Longfei Zhao, Qiang Li, Jiazheng Sun, Fanjun Chen, Qingchun Pan

**Affiliations:** 1 Key Laboratory of Plant-Soil Interactions of Ministry of Education, National Academy of Agriculture Green Development, College of Resources and Environmental Sciences, China Agricultural University, Beijing 100193, China; wubin_rush@163.com (B.W.); renwei2012@126.com (W.R.); zhaolongfei4992266@163.com (L.Z.); liqiang14329@cau.edu.cn (Q.L.); sjz2020cau@cau.edu.cn (J.S.); caucfj@cau.edu.cn (F.C.); 2 Sanya Institute, China Agricultural University, Sanya 572025, China

**Keywords:** maize, root system architecture, genome-wide association study, candidate gene

## Abstract

Roots are important plant organs for the absorption of water and nutrients. To date, there have been few genome-wide association studies of maize root system architecture (RSA) in the field. The genetic basis of maize RSA is poorly understood, and the maize RSA-related genes that have been cloned are very limited. Here, 421 maize inbred lines of an association panel were planted to measure the root systems at the maturity stage, and a genome-wide association study was performed. There was a strong correlation among eight RSA traits, and the RSA traits were highly correlated with the aboveground plant architecture traits (e.g., plant height and ear leaf length, *r* = 0.13–0.25, *p* < 0.05). The RSA traits of the stiff stalk subgroup (SS) showed lower values than those of the non-stiff stalk subgroup (NSS) and tropical/subtropical subgroup (TST). Using the RSA traits, the genome-wide association study identified 63 SNPs and 189 candidate genes. Among them, nine candidate genes co-localized between RSA and aboveground architecture traits. A further co-expression analysis identified 88 candidate genes having high confidence levels. Furthermore, we identified four highly reliable RSA candidate genes, *GRMZM2G099797*, *GRMZM2G354338*, *GRMZM2G085042*, and *GRMZM5G812926*. This research provides theoretical support for the genetic improvement of maize root systems, and it identified candidate genes that may act as genetic resources for breeding.

## 1. Introduction

Global agricultural production is facing its greatest challenge owing to climate change, land degradation and population growth [1,2]. Breeding stress-resistant and nutrient-efficient crops is necessary to assure food security [3]. Root architecture, including structural traits such as root width, depth, and length [4], determines the responsiveness and adaptability of plants to abiotic stress [5]. The ideal root system architecture (RSA) can efficiently obtain water and nutrient resources from soil, ensure the growth and development processes of plants, and improve productivity [6,7]. The genetic improvement of the RSA is necessary for increasing maize yields and is expected to further improve the water and nutrient utilization efficiency [8]. Root traits are hidden in the soil, and root growth cannot be directly observed, making it difficult to determine the phenotypes. To date, the potential of maize root trait improvement has not been fully exploited [9]. The identification of RSA traits and the mining of related genes are of great significance for the genetic improvement and increased adaptability of plants to the environment abiotic stresses.

Plant RSA has a strong plasticity, and it is affected by both environmental signals and genotype-associated internal mechanisms, which change in response to the external environment [5]. Root development is regulated by environmental signals, which affect the rate and direction of root growth [10]. For instance, maize responds to nitrogen stress by adjusting the root growth angle [11]. Steeper root angles increase the depth of the roots, thereby increasing the root distribution in the deep soil, which in turn allows roots to access water and nutrients that have leached into the deep-soil profile [7,11]. Under conditions of adequate water and nutrients, the root growth angle is relatively flat because the moisture and nutrients of the shallow soil profile meet the needs of aboveground growth [12]. In addition to typical RSA traits, such as angle and depth, lateral root length and density play important roles in plant nutrient uptake [7,13]. Under phosphorus deficiency conditions, plant RSA shows a shortening of primary roots and an increase in lateral roots, while root hairs grow in large quantities [14]. At present, optimal RSAs in different growth environments are a targets for root genetic improvement [7]. Furthermore, the development of the plant RSA is regulated by internal mechanisms [15,16,17].

Hormones are the main components, on the physiological level, of the internal regulatory mechanism of RSA [16]. Hormones, such as auxin, cytokinin, gibberellin, and ethylene, form complex hormonal networks in plants and participate in the development of RSA [15,18,19]. As an important indicator signal of the root system, auxin not only maintains root development, but also mobilizes many other hormones to participate in root regulation [17]. Several IAA-related genes regulate root architecture in *Arabidopsis* [20]. A study identified 81 genes involved in the plant hormone signal transduction pathway and 26 transcription factor genes that may regulate root growth [21]. Cytokinins are involved in the inhibition of lateral root formation through the involvement of auxin [22]. In addition, there are some non-hormonal signals, mainly metabolites, such as peptides, that regulate the development of RSA [23].

The RSA is a complex quantitative trait having wide phenotypic variation among different genotypes. Because root sampling is destructive and labor-intensive, there are limited studies on the genetic mechanisms of crop RSA in the field [24]. In recent years, with the development of computer science and modeling techniques for root phenotypic measurement [24], the analysis of the genetic basis of crop RSA has developed [25]. Research on gene mapping of the maize RSA is in the preliminary stage, and it is difficult to perform further fine mapping and to clone genes [26]. At present, the root genes reported in maize have been mainly cloned using mutants, including eight genes related to RSA development [27]. Genome-wide association studies (GWASs) have high mapping accuracies and do not require population construction, thereby providing a feasible method for studying the genetic basis of complex traits [28]. A GWAS of 22 seedling-stage root traits identified a root candidate gene, *GRMZM2G1537222*, which contains nine significant SNPs, most likely related to maize root development [29]. In addition, 17 SNPs significantly associated with root traits have been identified, and the gene *GRMZM2G02110*, which is specifically expressed in the root system, was located on chromosome 5 [30]. A previous study investigated 209 maize inbred lines using primary root length, seed root length, and seed root number at 35 days after germination under both normal water supply and drought conditions, and 7 root candidate genes were identified [31]. Moreover, under two different nitrate level treatments, 55 candidate genes significantly associated with 21 RSA and 3 aboveground traits were identified by GWAS [32]. In recent years, the construction of co-expression networks using gene expression data can be powerful for interpreting candidate genes through integrating GWAS results [33,34,35]. For instance, using this computational approach, candidate genes were prioritized from a large-scale GWAS examining the accumulation of 17 different elements in maize seeds [35]. In general, there are limited genetic-based analyses and candidate gene mining experiments of maize roots in the field, and a genetic analysis of RSA is needed to further understand its regulatory mechanisms.

To elucidate the genetic basis of maize RSA, we measured related traits at maturity under field conditions using an associated population of 421 inbred lines. Combined with the RSA phenotypes, we used high-density genotypes to perform a GWAS to investigate the genetic basis of maize RSA. Additionally, a co-expression analysis was performed using root transcriptome data to identify candidate genes related to maize RSA.

## 2. Materials and Methods

### 2.1. Experimental Materials and Design

The maize association panel used in this study included 421 maize inbred lines (Table S1) from China, the USA, and the International Maize and Wheat Improvement Center [36]. In accordance with a previous study, the 421 maize inbred lines were divided into four subgroups: SS, NSS, TST, and Mixed [36] (Table S1). Field experiments were conducted in 2015 at Nanbin Farm (18.18° N, 109.03° E) in Sanya, Hainan Province, China. Each inbred line was planted in a single row at a density of 100,000 plants ha^−1^ in a randomized block design. Each inbred line was planted in a 4 m single row, with a 50 cm row spacing and a 20 cm inter-plant spacing. There was a base application of compound fertilizer (nitrogen fertilizer, 90 kg N ha^−1^; phosphate fertilizer, 90 kg P_2_O_5_ ha^−1^; and potassium fertilizer, 90 kg K_2_O ha^−1^). Other field measures were implemented in accordance with conventional management practices.

### 2.2. Determination of Root Traits

At the mature stage, 3–5 plants of each inbred line with similar aboveground growth were selected and their root systems were excavated to a depth of 30 cm. The excavated roots were gently shaken to remove most of the attached soil and soaked in a water mixed with detergent. A washing apparatus with adjustable water pressure was then used to remove any remaining soil attached to the roots. After being cleaned, the root system was transferred to a studio with stable light-emitting diode (LED) light to collect two-dimensional images of the root systems using a camera (ILCE-5100, Sony, Tokyo, Japan). All the root images were stored in JPEG file format. The software DIRT was used for the high-throughput quantitative analysis of two-dimensional root images with a masking threshold = 20 [37]. In total, eight root traits related to root width, angle, and area were derived from the images (Table 1 and Table S2).

### 2.3. Data Organization and Statistical Analysis of Root Traits

The phenotypic data of aboveground traits used in this study, including plant height, ear position, and leaf traits, were derived from published studies [38] (Tables S3 and S4). A statistical analysis was performed using R4.03 software (https://www.r-project.org/, accessed on 10 August 2021), and the pearson correlation coefficient was calculated using R package “Hmisc”. The principal component analysis was performed using R package “ggbiplot”. On the basis of subgroup classifications, box plots of the eight root traits were constructed and compared using R package “boxplot”.

### 2.4. Genome-Wide Association Study and Candidate Gene Mining

The genotypes, population structure, and kinship matrix of the association panel used for the GWAS in this study were downloaded from MaizeGO (http://www.maizego.org/index.html, accessed on 8 June 2020). The genotypic data were compared with the reference genome B73 RefGen_v2 (https://www.maizegdb.org/, accessed on 8 June 2020). In this study, eight root traits and eight aboveground traits were used as phenotypes. The genome-wide association study was performed by TASSEL5.0 [28,39] using a mixed linear model with 1.25 million high-density SNP markers. Given the rigor of the mixed linear model, we conservatively chose −log_10_ (*p*-value) = 5.0 as the threshold for determining whether the marker was significantly associated with the root traits. Manhattan plots and a linkage disequilibrium heat map were constructed using R package “CMplot” and “LDheatmap”, respectively. Using B73 RefGen_v2 as the reference genome, root candidate genes were searched for in the regions 50-kb up- and downstream of significant SNPs. MaizeGDB (https://www.maizegdb.org, accessed on 16 July 2021), NCBI (https://www.ncbi.nlm.nih.gov, accessed on 16 July 2021), and TAIR (https://www.arabidopsis.org, accessed on 16 July 2021) databases were used to annotate candidate genes.

### 2.5. Construction and Analysis of Maize Root Co-Expression Networks

Camoco [35] was used to identify the high-priority candidate RSA-related genes in maize. The locus information used in co-expression analysis was derived from GWAS. The root transcriptome gene expression levels of 48 different maize inbred lines in published studies were used to construct the co-expression network [35]. The density metrics were used to conduct the co-expression network analysis. The candidate window size and maximum number of flanking genes were 50 kb and two, respectively. The candidate windows were determined both upstream and downstream of the input SNPs. Cytoscape [40] was used to visualize the network with a highly prioritized gene set.

### 2.6. Root Transcriptome Sequencing and Expression Analysis

In total, 83 inbred lines of the association panel were randomly selected and planted in the Shangzhuang Experimental Station of the China Agricultural University in 2019. Then, 2–3 plants per line were selected at the silking stage, and two layers of roots below the soil surface were collected and frozen in liquid nitrogen. TRIzol reagent (TaKaRa, Tokyo, Japan) was used extract total RNA in accordance with the manufacturer’s instructions. RNA integrity was assessed using the RNA Nano 6000 Assay Kit and the Agilent Bioanalyzer 2100 system (Agilent Technologies, Santa Clara, CA, USA). Sequencing libraries were generated using an NEBNext Ultra RNA Library Prep Kit for Illumina (NEB, Ipswich, MA, USA). Libraries were sequenced on an Illumina HiSeq 6000 system. HISAT2 [41] was used for the sequence alignment, and StringTie [42] was used to assemble and quantify gene expression levels into FPKM (fragments per kilobase of exon model per million mapped fragments) values after the alignment analysis was completed. The FPKM value was used to compare the differences in gene expression levels between the corresponding inbred lines having different haplotypes.

## 3. Results

### 3.1. Phenotypic Variation of Root Traits

**Figure 1:**
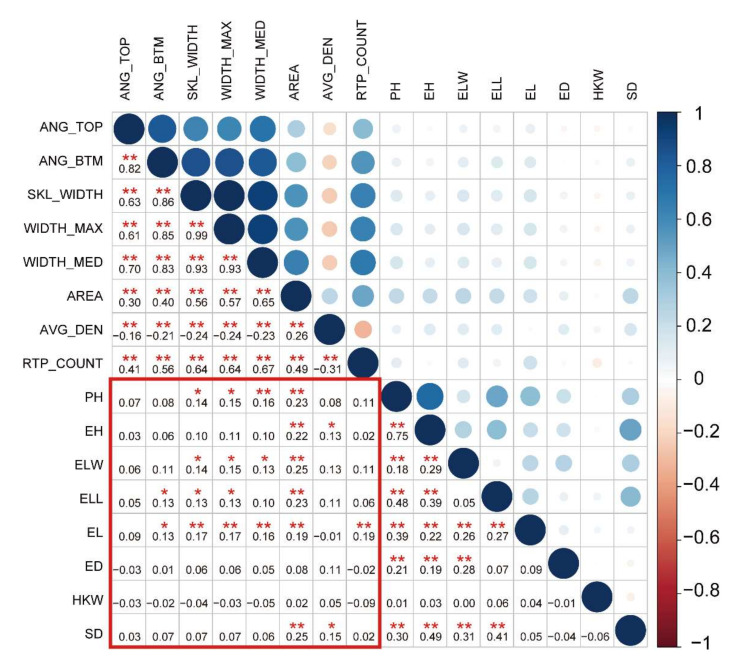
Pearson’s correlation analysis of root and aboveground traits. The color and size of a circle reflects the value of the correlation coefficient. The numbers form the correlation coefficient matrix. * used to mark significance: * represents *p* < 0.05 and ** represents *p* < 0.01.

The eight root traits measured in this study were root top angle (ANG_TOP), root bottom angle (ANG_BTM), root skeleton width (SKL_WIDTH), the maximum width of the root system (WIDTH_MAX), the median width of the root system (WIDTH_MED), projected root area (AREA), average root density (AVG_DEN) and the number of root tip paths (RTP_COUNT) (Table 1). The detailed information regarding these traits can be found in Table 1. The eight RSA traits showed abundant genetic variation, with variation coefficients ranging from 0.068 to 0.560 (Table 1). The phenotypic values showed an approximately normal distribution (Figure S1). This indicated that the eight RSA traits were typical quantitative traits. The correlation analyses of the eight root traits showed that AVG_DEN was negatively correlated with the other seven root traits, except AREA (*r* = −0.31–−0.16; *p* < 0.05; Figure 1). There were significant positive correlations among the other six root traits (*r* = 0.26–0.99, *p* < 0.05; Figure 1). Notably, the maximum root width (WIDTH_MAX), mean root width (WIDTH_MED), and root skeleton width (SKL_WIDTH) were significantly correlated (*r* = 0.93–0.99, *p* < 0.01; Figure 1), which suggests similar regulatory mechanisms between WIDTH_MAX, WIDTH_MED, and SKL_WIDTH. A principal component analysis showed that the first principal component could explain 99.9% of the variation. Thus, among the eight root traits, traits related to root width, growth angle, and number of roots explained most of the root phenotypic variation (Figure S2). In addition, the correlation analysis showed that root traits were positively correlated with aboveground agronomic traits (*r* = 0.13–0.25, *p* < 0.05, Figure 1). For example, there was a correlation between AREA and ear leaf length. Furthermore, the correlation between RSA traits and aboveground yield traits was not significant (*p* > 0.05; Figure 1). Thus, in terms of phenotype, there is a potential similar mechanism between root and aboveground agronomic traits. Additionally, it revealed that the formation of yield traits is complicated, and the formation is determined by a variety of factors, including roots.

### 3.2. Analysis of Root Architecture Variation in Different Maize Subgroups

**Figure 2:**
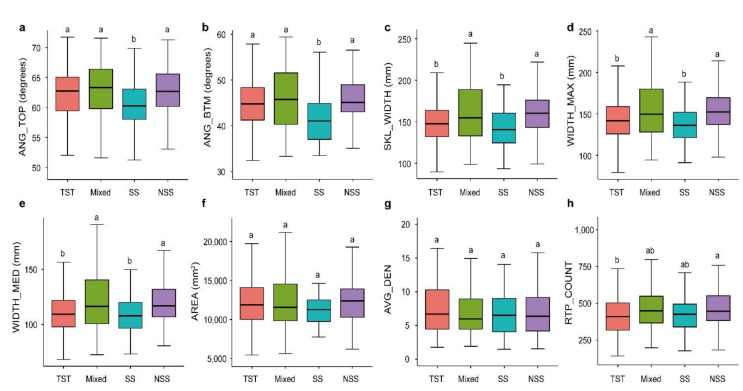
Comparison of root traits among different maize subgroups. Differences in (**a**) AREA, (**b**) WIDTH_MED, (**c**) ANG_TOP, (**d**) SKL_WIDTH, (**e**) AVG_DEN, (**f**) WIDTH_MAX, (**g**) ANG_BTM and (**h**) RTP_COUNT among TST, NSS, SS and Mixed subgroups of maize inbred lines. Letters above the plots indicate significant differences between different subgroup according to a one-way ANOVA and Duncan’s multiple comparisons. Four subgroups: Stiff stalk (SS), Non-stiff stalk (NSS), Tropical/subtropical (TST), and Mixed group (Mixed). Different lowercase letters represent significant differences among four maize subgroups (*p* < 0.05).

To investigate the variations in RSAs of different germplasm resources, we analyzed the variances of root traits among different groups. Overall, compared with Non-Stiff Stalk (NSS), Mixed group (Mixed), and Tropical/subtropical (TST) subgroups, the Iowa Stiff Stalk Synthetic (SS) subgroup had lower root trait values (Figure 2a–e). Our study found that there were no significant differences in AREA and AVG_DEN among the four subgroups (*p* > 0.05, Figure 2f,g). Among the ANG_TOP and ANG_BTM traits related to root growth angle, the SS subgroup showed a significant lower value than the other three subgroups (*p* < 0.05, Figure 2a,b). For WIDTH_MED, WIDTH_MAX, and SKL_WIDTH traits, the TST and SS subgroups showed significant higher values than the Mixed and SS subgroups (*p* < 0.05, Figure 2c–e). For the RTP_COUNT trait, the TST and NSS subgroups showed significant differences (*p* < 0.05, Figure 2h). The above results suggested that the SS subgroup had a significant less complicated RSA compared with those of the other subgroups, which was manifested by a steeper and narrower root system. The results of this study provide important guidance for maize root genetic improvement.

### 3.3. Genome-Wide Association Analysis of Root Traits

**Figure 3:**
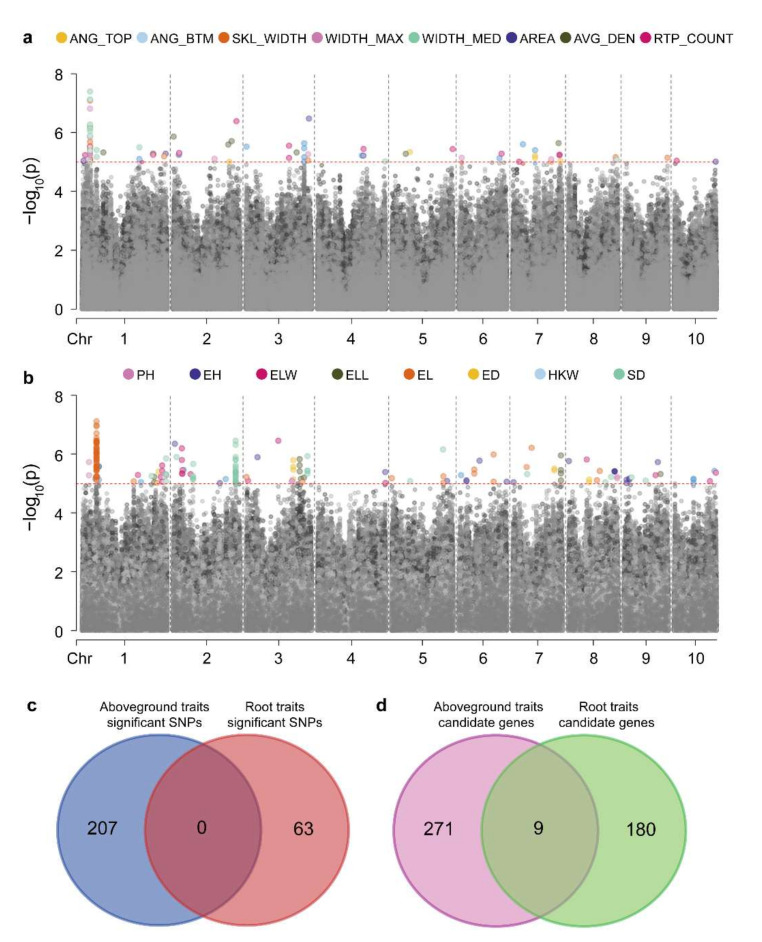
Genetic correlation between aboveground and root traits. Manhattan map of genome-wide association analysis of (**a**) root and (**b**) aboveground traits, the red dotted line shows the threshold (LOD = 5), points below the threshold is gray. (**c**) SNPs co-located using root and aboveground traits. (**d**) Genes co-located using root and aboveground traits.

We performed a GWAS on eight RSA traits in the associated population and identified 63 significantly associated SNPs (Figure S3 and Table S5). We found multiple traits mapped to the same SNP locus (Figure 3a), because close correlations between maize RSA traits were identified (Figure 1). Using B73 RefGen_v2 as the reference genome, candidate genes were searched for in regions 50-kb upstream and downstream of these significant loci, and 189 candidate genes were identified (Table S6). The co-mapping of different root traits to the same loci suggested that the genes controlling maize RSA have multiple effects. For example, *GRMZM2G099797*, co-localized with RTP_COUNT, WIDTH_MAX, WIDTH_MED, and SKL_WIDTH (Table S6), may affect maize root development by regulating cytokinin [43].

Additionally, the mapping results of root and aboveground traits were compared. From the aboveground agronomic and yield traits, we identified 207 significant SNPs and 280 candidate genes (Figure 3b, Figure S4 and Table S7). There were no significant SNPs identified between roots and the agronomic and yield traits (Figure 3c). However, nine common candidate genes were identified between RSA traits and aboveground traits (Figure 3d, Tables S6 and S8). In our study, potential candidate genes for root architecture were identified, and genetic differences between aboveground agronomic traits and RSA traits were revealed.

### 3.4. Root Candidate Gene Mining Combined with the GWAS and Co-Expression Analysis

**Figure 4:**
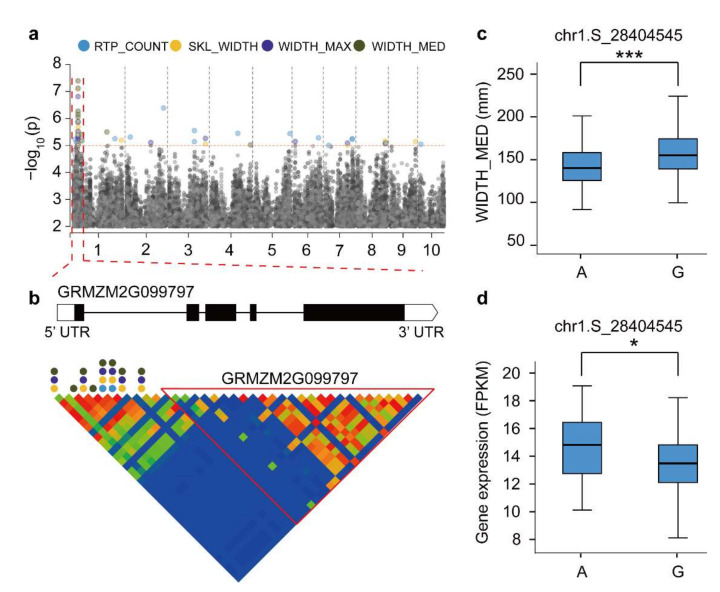
Linkage analysis of *GRMZM2G099797* gene regions and a haplotype material analysis. (**a**) Manhattan plot of four associated traits. (**b**) *GRMZM2G099797* genetic structure and linkage analysis of gene regions. The circular dots represent SNPs associated with root traits. (**c**) Differences in WIDTH_MED among various haplotype materials. (**d**) Differences in gene expression level between different haplotype materials. * Used to mark significance as determined by a t-test: * represents *p* < 0.05, *** represents *p* < 0.001.

To further identify high confidence candidate genes involved in maize RSA, we performed a co-expression analysis in combination with root RNA-seq and a GWAS. Among 189 candidate genes from the GWAS, 88 for root traits were identified as high-priority genes by the gene co-expression analysis (Figure S5 and Table S9). We found that *GRMZM2G099797* was associated with WIDTH_MED, WIDTH_MAX, SKL_WIDTH, and RTP_COUNT traits simultaneously (Figure 4a,b). This gene encodes a cytokinin-response regulator, which is highly expressed in the root system. Its *Arabidopsis*, the expression of the homolog *AT3G16857* reduces the effects of cytokinin inhibition on root elongation and lateral root formation [44]. A haplotype analysis of significant SNPs (chr1.S_28404545) revealed significant phenotypic and gene expression differences associated with root architecture (Figure 4c,d). Two candidate genes (*GRMZM2G354338* and *GRMZM2G085042*) were associated with SNP markers (chr6.S_142873590) corresponding to ANG_BTM (Figure S6a). By comparing the haplotypes, we found significant differences in ANG_BTM between A/T and T/C (*p* < 0.001; Figure S6b). In addition, the expression levels of different haplotypes of *GRMZM2G354338* and *GRMZM2G085042* showed significant differences (*p* < 0.05 and *p* < 0.01, respectively; Figure S6c). As a maize-specific gene, the GRMZM2G354338-encoded *ZmARGOS8* is a member of the *ARGOS* gene family, which contains a conserved TPT domain that mediates the formation of maize node roots by modulating sensitivity to ethylene [45]. The function of *GRMZM2G085042* in maize has not been studied, and its corresponding *Arabidopsis* homolog *AT4G01220* plays an important role in the root development [46]. Furthermore, we identified the gene *GRMZM5G812926* as being associated with RTP_COUNT (Figure S6d). The trait RTP_COUNT among different haplotypes was significantly different (*p* < 0.001, Figure S6e), but no significant difference in gene expression was determined (*p* = 0.51, Figure S6f). In general, this study provides important candidate gene resources for future maize root genetic improvements.

## 4. Discussion

Maize originated in south-central Mexico and has been improved by natural and artificial selection for a long time [47]. During domestication and improvement, maize cultivation spread from tropical to temperate regions. In the process of adapting to different environments, the production efficiency of maize also increased significantly [48,49]. As an important organ for water and nutrient absorption, roots have been neglected owing to the difficulty in determining phenotypes. In recent years, the importance of root traits has been gradually accepted, and the utilization of root traits is expected to lead to a “second green revolution” in agriculture [50]. Most of the adaptive traits in maize have been selected and fixed during evolution and adaptation in tropical to temperate climates [51]. During the processes of evolution and improvement, genomic differentiation and selection between the TST germplasm and temperate germplasm have emerged, and root traits and genomic loci may have been selected [52]. The 421 inbred lines used in this study showed abundant genetic diversity and *r* values representing global maize diversity [36]. The samples were divided into four subgroups, SS, NSS, TST, and Mixed. SS and NSS are temperate germplasm resources, and TST is a tropical/subtropical germplasm resource. Compared with the other three subgroups, the SS subgroup had steeper root angles and narrower root widths (Figure 2). During the selection of germplasm resources, unlike the genomes of the NSS and TST subpopulations, those of the SS subpopulation had narrow genetic backgrounds, and the differences between the root traits of the SS subpopulation and those of the other subpopulations were due to long-term adaptation and selection [36]. Therefore, we speculated that the SS subgroup was selected for genes associated with narrow RSA during genomic differentiation, and these genes were retained during maize breeding.

The root system forms an important organ for plants to absorb water and nutrients from the soil, which plays an important role in ensuring the normal growth and development of plant aboveground parts. For example, the growth and development of plant buds is influenced by root hormones, whereas nutrient absorption by the root system is regulated by stem-derived substances [53]. In this study, we found that there was a high correlation between aboveground agronomic and root traits, especially RSA and aboveground plant-type traits (Figure 1). However, the genetic correlation between aboveground and root systems in plants is still poorly understood. A further GWAS of RSA traits and aboveground traits identified nine shared genes (Figure 3). These results suggested that although there was a strong physiological correlation between RSA traits and aboveground agronomic traits, there are significant genetic differences between them. At present, root traits have been identified based on quantitative trait locus (QTL) mapping. However, most of this has been primary mapping [26,27]. Furthermore, previous studies mainly focused on root traits at the seedling stage, which do not reflect the true RSA of maize under field conditions. On the basis of GWASs, genetic mechanism analyses and gene mining of maize root traits have been performed, but few genes related to maize RSA have been identified [25]. In this study, a GWAS was conducted for maize RSA traits under field conditions, and 189 root candidate genes were identified (Table S6). However, the GWAS produced some inevitable false positives owing to the large number of multiple tests [54]. To more efficiently identify high-priority RSA-related candidate genes, a gene co-expression network was constructed using root transcriptome data. Based on the root co-expression network analysis, we identified 88 high-priority candidate genes (Table S9), which provide valuable genetic resources for maize root genetic improvement.

Plant hormones play important roles in regulating the development of plant organs [16]. Auxins play leading roles in regulating root development, and several auxin pathway genes have been cloned [55]. In addition, cytokinins play important roles in regulating root systems [56]. They affect root trait development by influencing the interactions between auxins and cytokinins [15]. In this study, we identified a candidate gene *GRMZM2G099797* (*ZmCRR8*) (Figure 4), which encodes a cytokine-response regulator [57]. Cytokinin-response regulators are composed of A- and B-type response regulators [58]. This gene family plays roles in plant root tropism [59] and abiotic stress response [60]. Its regulation may be related to the coordinated control of root growth and development by auxins [43]. In a previous study, *ZmCRR8* activated the expression of the maize *ZmWUS* gene and regulated the development of inflorescence meristem [61]. The overexpression of B-type cytokinin response regulators in *Arabidopsis* leads to the significant inhibition of root development, which may be caused by the disruption of cytokinin homeostasis [57]. Thus, we speculated that the high expression of *ZmCRR8* may induce the expression of cytokinins in roots, leading to disordered hormone levels in roots, ultimately inhibiting root development.

Furthermore, ethylene plays an important role in regulating plant development [62]. The *GRMZM2G354338* (*ZmARGOS8*) gene identified in this study (Figure S6),encodes a negative regulator of ethylene reactions [63]. *ZmARGOS8*-overexpression plants show ethylene insensitivity and significantly reduced responses of roots induced by ethylene [64]. With the increased expression of *ZmARGOS8*, ANG_BTM increased (Figure S6a), which may result from the expression inhibiting the promotive effect of ethylene on lateral root formation [64]. The overexpression of *ZmARGOS8* improves maize yield under drought-stress conditions [64], and it also delays the development of maize nodal roots and increases maize yields [45]. This suggests that *ZmARGOS8* regulates not only the development of maize roots, but also the development of the aboveground parts. Thus, it has a high breeding potential. In addition, *GRMZM2G085042* was significantly associated with ANG_BTM (Figure S6a). The function of this gene in maize has not been reported. Its *Arabidopsis* homolog *AT4G01220* (*MGP4*) encodes a rhamnogalacturonan II xylosyltransferase, and its expression results in significant root defects and partial plant death [46]. Furthermore, *GRMZM5G812926*, which is significantly associated with RTP_COUNT (Figure S6d), may be involved in the regulation of maize root development. Its *Arabidopsis* homolog *AT1G30440* (*NPH3*), which encodes a protein having an NPY3 domain [65]. This domain may play an essential role in auxin-mediated organogenesis and root gravitropic responses in *Arabidopsis* [65]. Therefore, we think that four genes (*GRMZM2G099797*, *GRMZM2G354338*, *GRMZM2G085042*, and *GRMZM5G812926*) identified in this study are worthy of attention as RSA-related candidate genes.

Overall, owing to the difficulty and inefficiency of root trait identification, breeders tend to ignore the genetic analysis and improvement of root traits and focus instead on aboveground traits. With the continuous development of computer science and image recognition technology, the throughput acquisition of root phenotypes has been significantly increased. Using a high-throughput root phenotypic determination method, our study acquired mature maize root phenotypes, which laid an important foundation for the analysis of maize root genetic mechanisms. To date, we have a very limited understanding of the genes that control maize root development, and no major RSA genes that affect maize breeding have been found [26]. With the development of high-throughput root phenotypic technology and various gene editing techniques [66], it has become a reality to directly select root traits for crop phenotypic improvement [27]. This study revealed candidate genes and possible molecular mechanisms for regulating RSA, providing important insights and gene resources for the efficient breeding of maize with genetically improved RSA.

## 5. Conclusions

In this study, an association panel consisting of 421 maize inbred lines was used to measure RSA traits at the maturity stage under field conditions. First, we found that, compared with other germplasms, the SS germplasm has a narrower and steeper RSA. We then identified 189 candidate genes using a GWAS. In addition, we found that there were significant genetic differences between the root and the aboveground agronomic traits of maize. Finally, we conducted a co-expression analysis using root transcriptome data and identified 88 high-confidence candidate genes. We then identified four potential RSA-related candidate genes on the basis of gene function annotation. The results of this study provide valuable resources for the future genetic improvement of maize RSA.

## Figures and Tables

**Table 1:** Descriptions of eight root architecture traits and their statistical data. Max: maximum; Min: Minimum; CV: Coefficient of Variation.

Trait	Description	Unit	Max	Min	Mean	STDEV	CV
ANG_TOP	Root top angle	degrees	71.7	49.1	62.4	4.3	0.068
ANG_BTM	Root bottom angle	degrees	59.4	29.8	44.9	5.6	0.126
SKL_WIDTH	Root skeleton width	mm	253	89.6	155.2	29.7	0.191
WIDTH_MAX	Maximum width of the root system	mm	242.8	79.3	148.8	29.1	0.196
WIDTH_MED	Median width of the root system	mm	190.6	68.2	115.2	21.7	0.188
AREA	Projected root area	mm^2^	22,132	4828.9	12,219.5	3184.8	0.261
AVG_DEN	Average root density	-	29.3	1.5	7.4	4.2	0.560
RTP_COUNT	Number of root tip paths	count	1044	126.7	442.9	148.2	0.335

## Data Availability

Data supporting the findings of this work are available within the paper and its Supplementary Information.

## Supplementary Materials

The following supporting information can be downloaded at: https://www.mdpi.com/article/10.3390/genes13020181/s1. Figure S1: Histogram of distribution of eight root traits, Figure S2: Principal component analysis of root and aboveground traits, Figure S3: Manhattan plot of eight root traits analyzed by GWAS, Figure S4: Manhattan map of aboveground traits analyzed by GWAS, Figure S5: Co-expression network analysis of root candidate genes, Figure S6: GWAS identification of high-priority genes for variation in maize root traits, Table S1: Information from the maize lines in the panel, including the pedigree and subpopulation, Table S2: The phenotypic data of eight root traits, Table S3: Description of eight aboveground traits, Table S4: The phenotypic data of aboveground traits used in this study, Table S5: The number of significant SNPs and candidate genes associated with eight root traits, Table S6: SNPs significantly associated with eight root traits and the candidate genes within 50 kb up- and downstream of each unique significant association SNPs, Table S7: The number of significant SNPs and candidate genes associated with eight aboveground traits, Table S8: SNPs significantly associated with eight aboveground traits and the candidate genes within 50 kb up- and downstream of each unique significant association SNPs, and Table S9: List of high-priority candidate genes generated from co-expression network analysis.

## Abbreviations

GWAS	Genome-wide association study
Mixed	Mixed subgroup
NSS	Non-stiff stalk subgroup
RSA	Root system architecture
SNP	Single nucleotide polymorphism
SS	Stiff stalk subgroup
TST	Tropical/subtropical subgroup
