# Supplementary material for: Genome-Wide Association Study of Root System Architecture in Maize
Source: Genes (Basel). 2022 Jan 28;13(2):181. doi: 10.3390/genes13020181 (PMC8872597; doi:10.3390/genes13020181)
Supplement: Supplementary file 1 [file genes-13-00181-s001.zip › Supplementary Figure Legends.pdf]

Figure legends of Supplementary Figures

Figure S1. Histogram of distribution of eight root traits.
The X axis represents the value of root traits and the Y axis represents density. The red line is the normal distribution fitting line.

Figure S2. Principal component analysis of root and aboveground traits.
(**a**) AREA and RTP_COUNT fall outside the confidence interval. (**b**) Principal component analysis of root and aboveground traits excludes AREA and RTP_COUNT. The X and Y axes represent the values of PCA1 and PCA2 in the transformed eigenvectors, respectively. The dotted ellipse represents a 95% confidence interval.

Figure S3. Manhattan plot of eight root traits analyzed by GWAS.
The red dotted line shows the threshold (LOD = 5), points above the threshold are marked red.

Figure S4. Manhattan map of aboveground traits analyzed by GWAS.
The red dotted line shows the threshold (LOD = 5), points above the threshold are marked red.

Figure S5. Co-expression network analysis of root candidate genes.
Each node represents a candidate gene, lines between nodes indicate the co-expression between two genes. The red hexagons highlight four high-priority candidate genes that are predicted to be strongly related to maize root development.

Figure S6. GWAS identification of high-priority genes for variation in maize root traits.
(**a**) GWAS identification of *GRMZM2G354338* and *GRMZM2G085042* as candidate genes for RSA variation. A partial Manhattan plot (upper) and an LD heat map (lower) are arranged. (**b**) The root trait of different haplotypes (chr6.S_142873590). (**c**) The expression levels of *GRMZM2G354338* and *GRMZM2G085042* between different haplotypes (box plot). (**d**) GWAS identification of *GRMZM5G812926* as candidate gene for RSA variation. A partial Manhattan plot (upper) and an LD heat map (lower) are arranged. (**e**) The root trait of different haplotypes (chr1.S_251385242). (**f**) The expression level of *GRMZM5G812926* between different haplotypes (box plot). Manhattan plot shows SNP associated with genes, star indicates significant SNPs loci. * used to mark significance: * represents $P < 0.05$ and ** represents $P < 0.01$.When no significant, the P value is displayed.
