# Supplementary figures and images for: Genome-Wide Association Study of Root System Architecture in Maize
Source: Genes (Basel). 2022 Jan 28;13(2):181. doi: 10.3390/genes13020181 (PMC8872597; doi:10.3390/genes13020181)

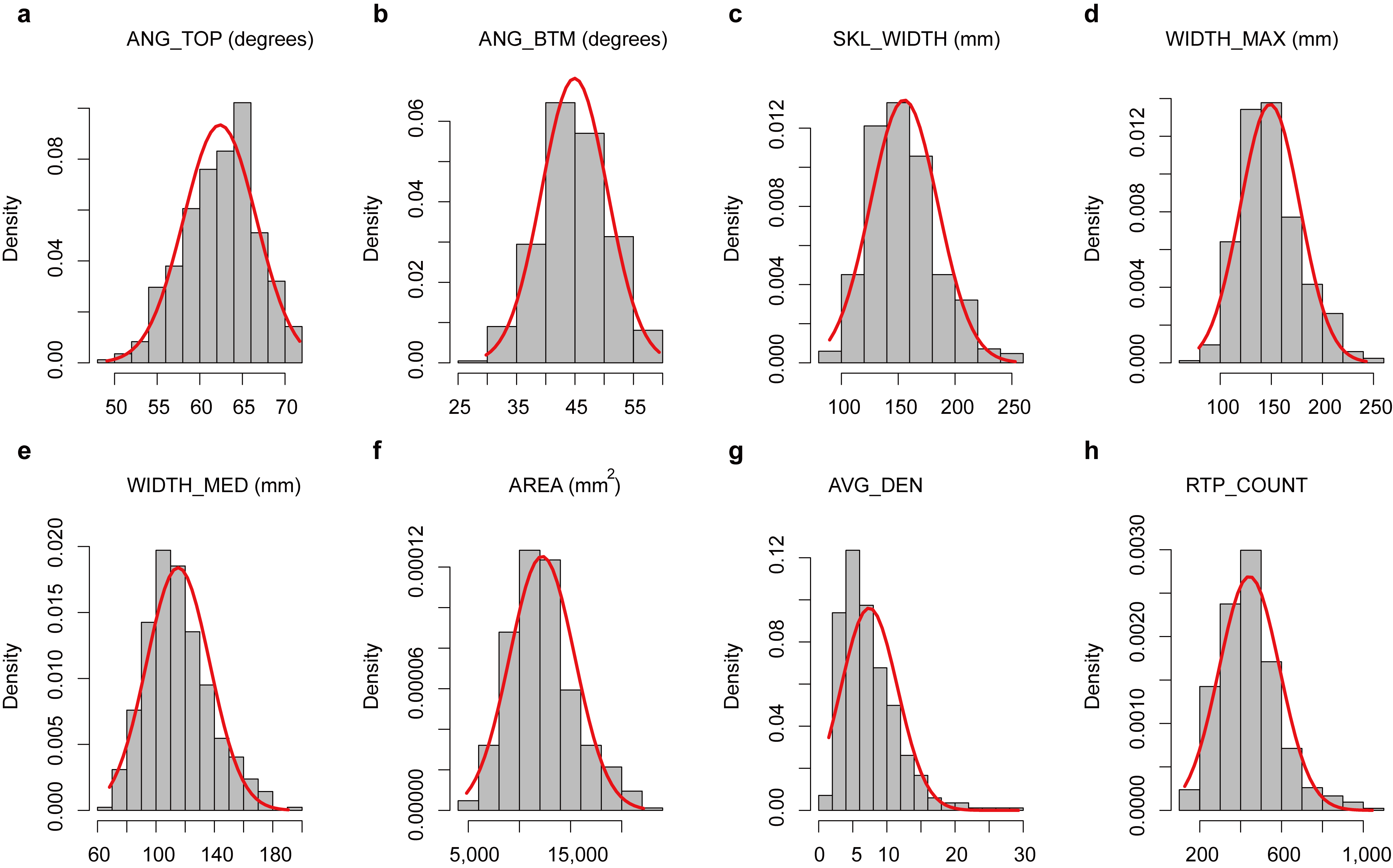

Supplement: Supplementary file 1 [file genes-13-00181-s001.zip › Figure S1.tif]

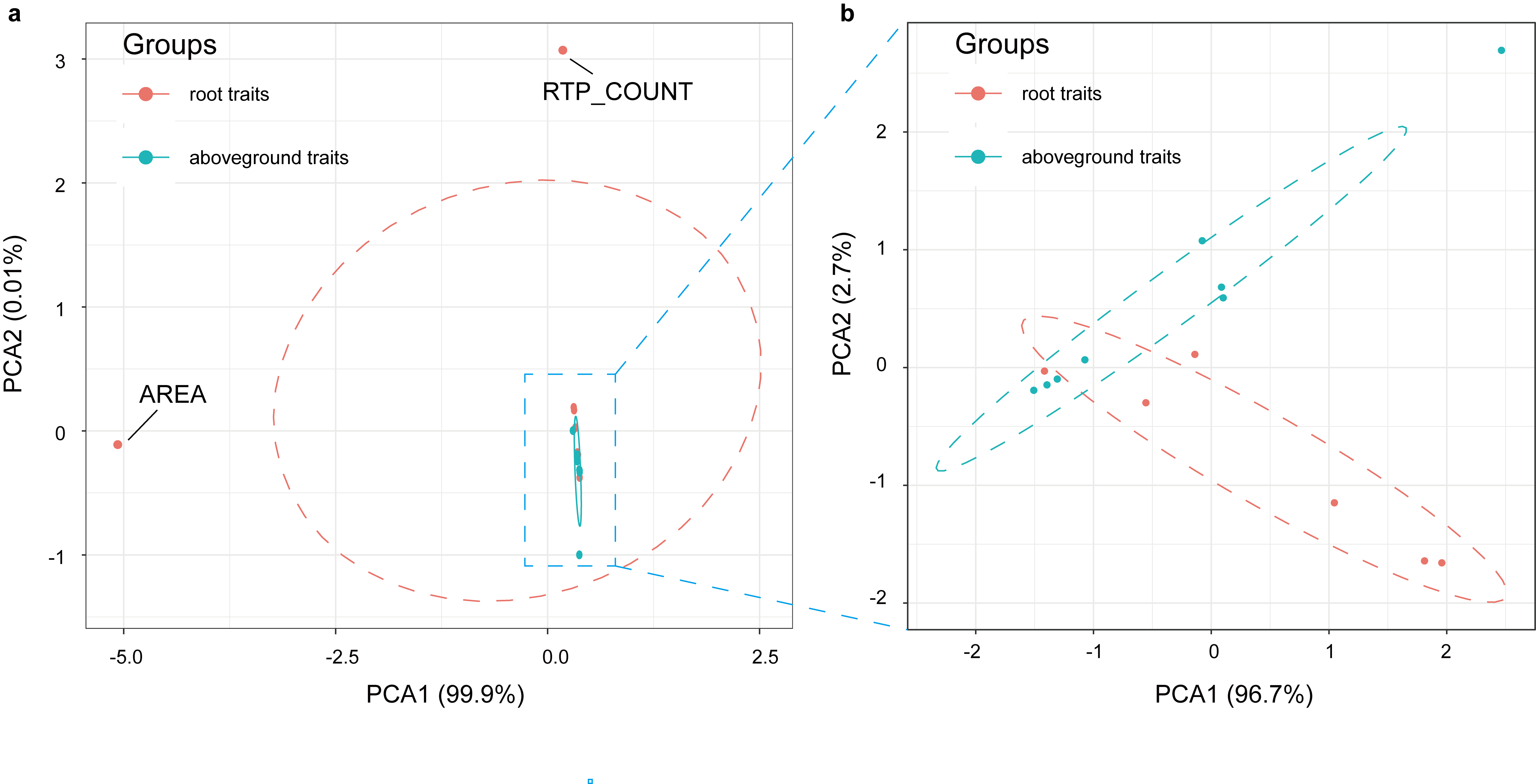

Supplement: Supplementary file 1 [file genes-13-00181-s001.zip › Figure S2.tif]

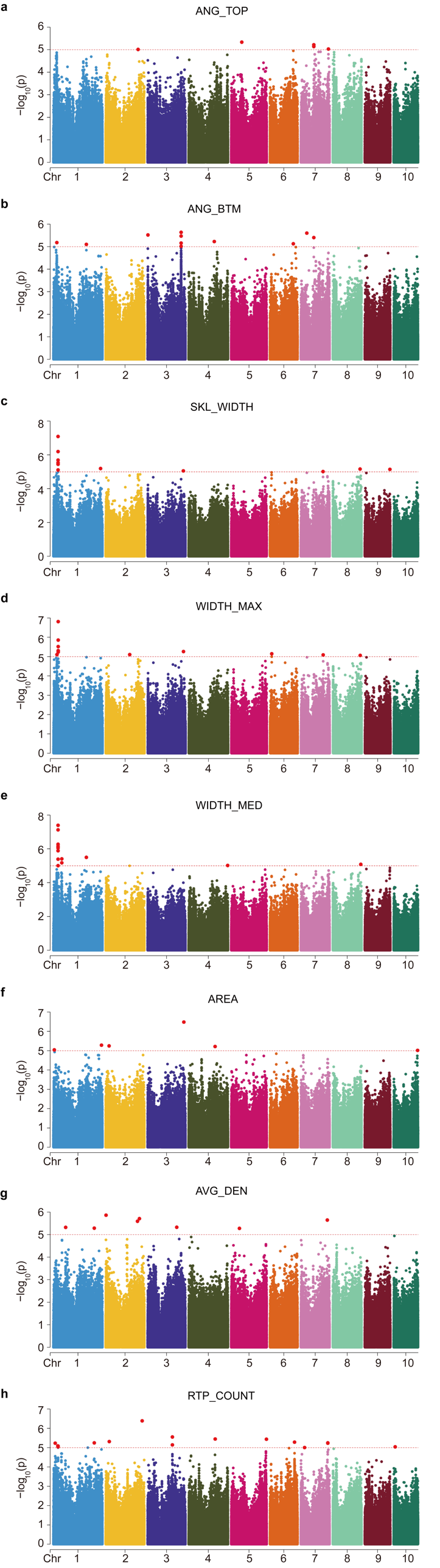

Supplement: Supplementary file 1 [file genes-13-00181-s001.zip › Figure S3.tif]

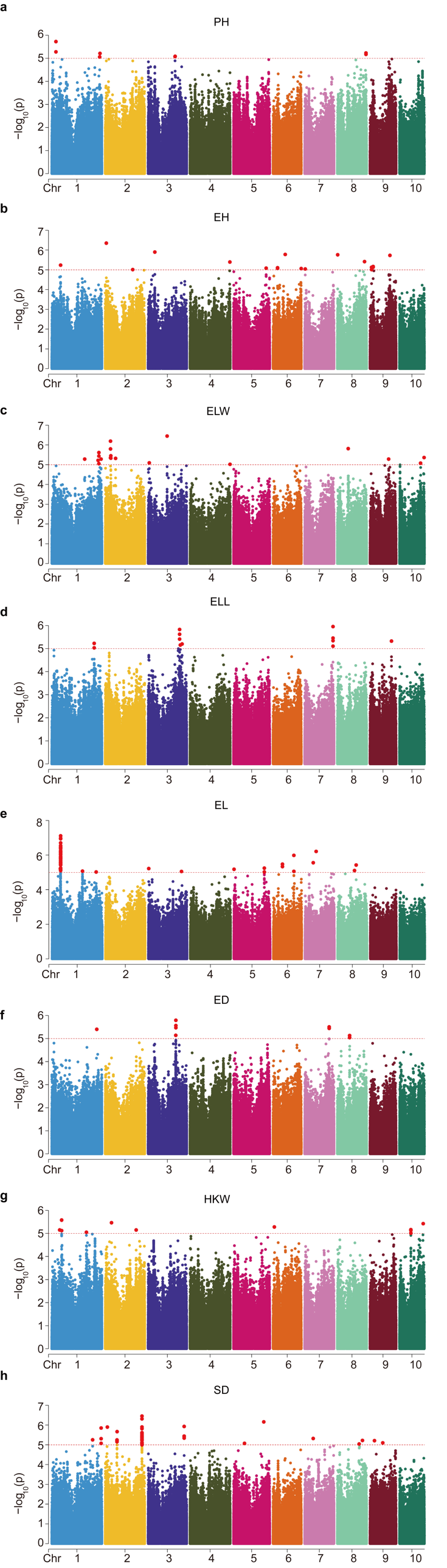

Supplement: Supplementary file 1 [file genes-13-00181-s001.zip › Figure S4.tif]

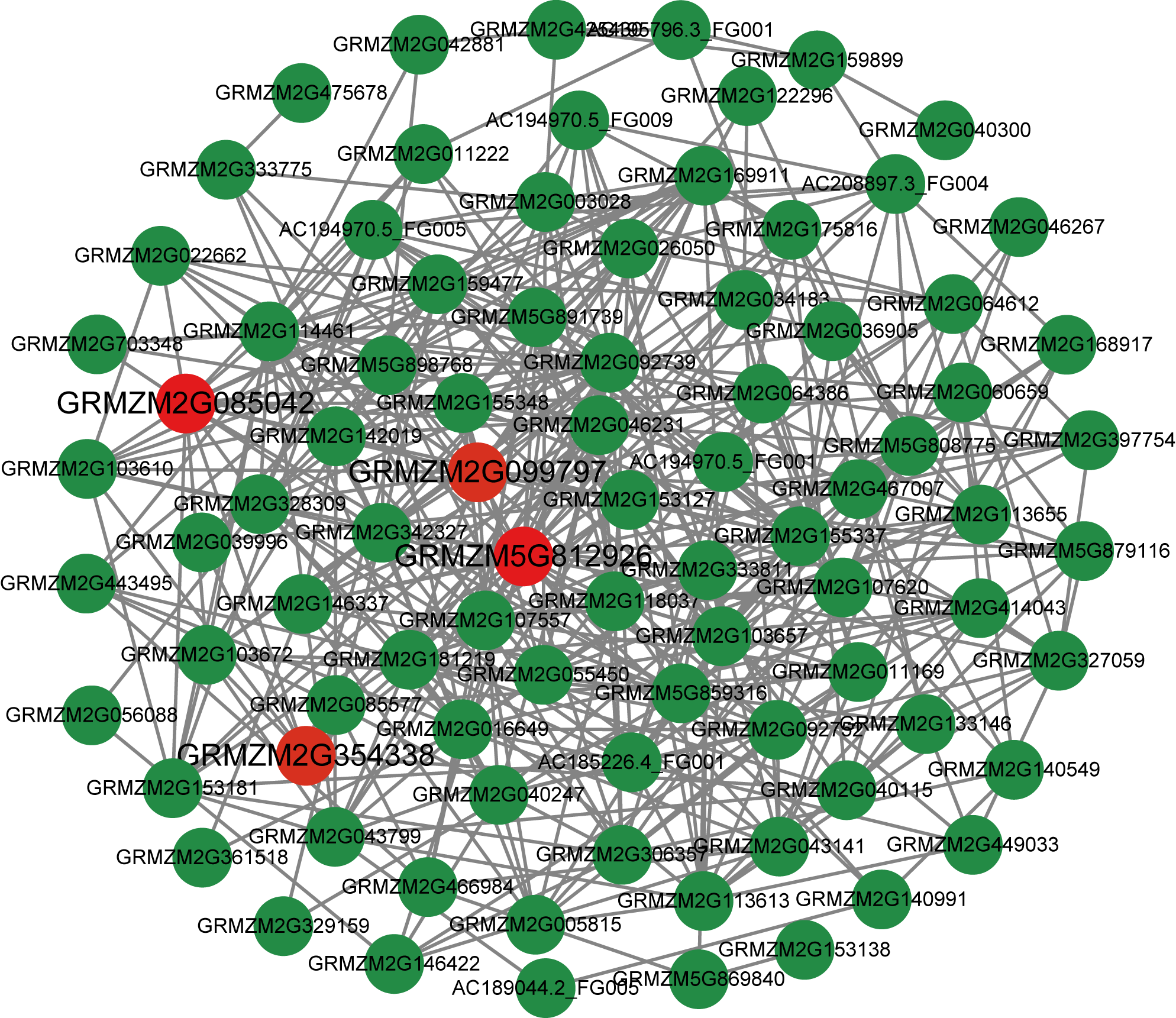

Supplement: Supplementary file 1 [file genes-13-00181-s001.zip › Figure S5.tif]

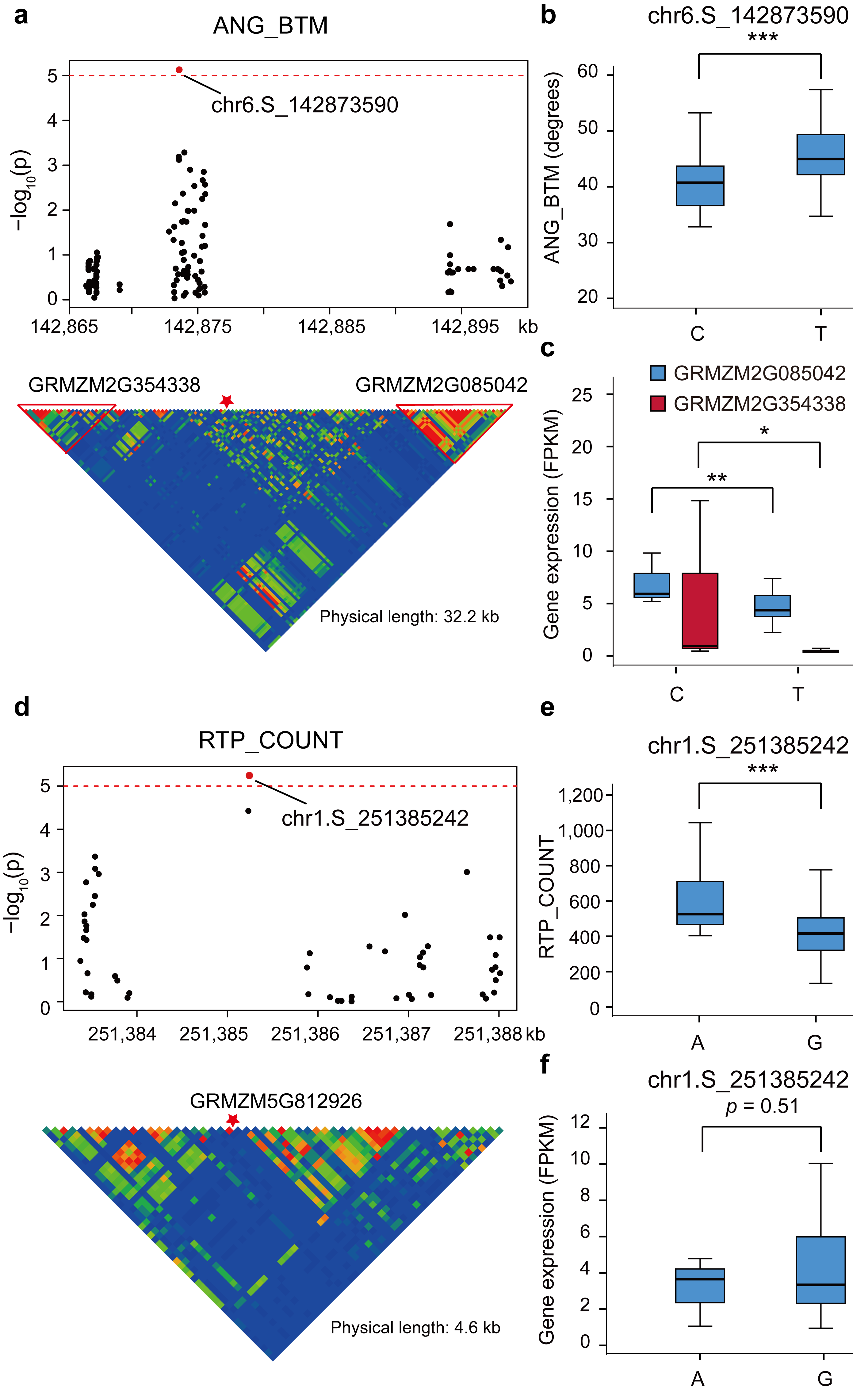

Supplement: Supplementary file 1 [file genes-13-00181-s001.zip › Figure S6.tif]
